# Public Health Risks, Dermatological Manifestations, and Environmental Justice Associated With Vinyl Chloride Exposure: Narrative Review

**DOI:** 10.2196/48998

**Published:** 2023-09-07

**Authors:** Rachel S Goodman, Lavanya Mittal, Eva Rawlings Parker

**Affiliations:** 1 School of Medicine Vanderbilt University Medical Center Nashville, TN United States; 2 The Ronald O Perelman Department of Dermatology Grossman School of Medicine New York University New York, NY United States; 3 Department of Dermatology Vanderbilt University Medical Center Nashville, TN United States; 4 Center for Biomedical Ethics and Society Vanderbilt University Medical Center Nashville, TN United States

**Keywords:** vinyl chloride, cutaneous manifestations, dermatology, industrial accident, climate advocacy, public health, environmental toxins, environmental health, acute, chronic, utilization, malignancy, community, socioeconomic, hazardous chemicals, toxicology

## Abstract

**Background:**

Environmental vinyl chloride (VC) exposure may result in serious acute and chronic dermatological conditions. Because existing literature largely focuses on exposures in occupational settings, a gap persists in our understanding of the medical consequences of large-scale chemical spills.

**Objective:**

This study aims to examine the potential dermatological manifestations of VC exposure in the context of industrial spills and other environmental disasters and to highlight the public health and justice implications of such releases.

**Methods:**

In this narrative review, relevant evidence-based, peer-reviewed scientific sources, gray literature, and media reports were identified via searches of search PubMed and Google using predetermined keyword search terms related to VC, VC spills and releases, train derailment, cutaneous disease, public health, and vulnerable and marginalized populations.

**Results:**

Contact dermatitis and frostbite may arise acutely, highlighting the importance of swift decontamination. Long-term manifestations from chronic VC exposure due to persistence in environmental reservoirs include Raynaud disease, sclerodermatous skin changes, acro-osteolysis, and cutaneous malignancies. The clinical severity of cutaneous manifestations is influenced by individual susceptibility as well as duration, intensity, and route of exposure. Additionally, chemical releases of VC more frequently impact Communities of Color and those of lower socioeconomic status, resulting in greater rates of exposure-related disease.

**Conclusions:**

With environmental release events of hazardous chemicals becoming increasingly common and because the skin has increased contact with environmental toxins relative to other organs, an urgent need exists for a greater understanding of the overall short- and long-term health impacts of large-scale, toxic exposures, underscoring the need for ongoing clinical vigilance. Dermatologists and public health officials should also aim to better understand the ways in which the disproportionate impacts of hazardous chemical exposures on lower-income and minority populations may exacerbate existing health disparities. Herein, we describe the health implications of toxic releases with particular consideration paid to marginalized and vulnerable populations. In addition to legal and regulatory frameworks, we advocate for improved public health measures, to not only mitigate the risk of environmental catastrophes in the future, but also ensure timely and effective responses to them.

## Introduction

**Figure 1:**
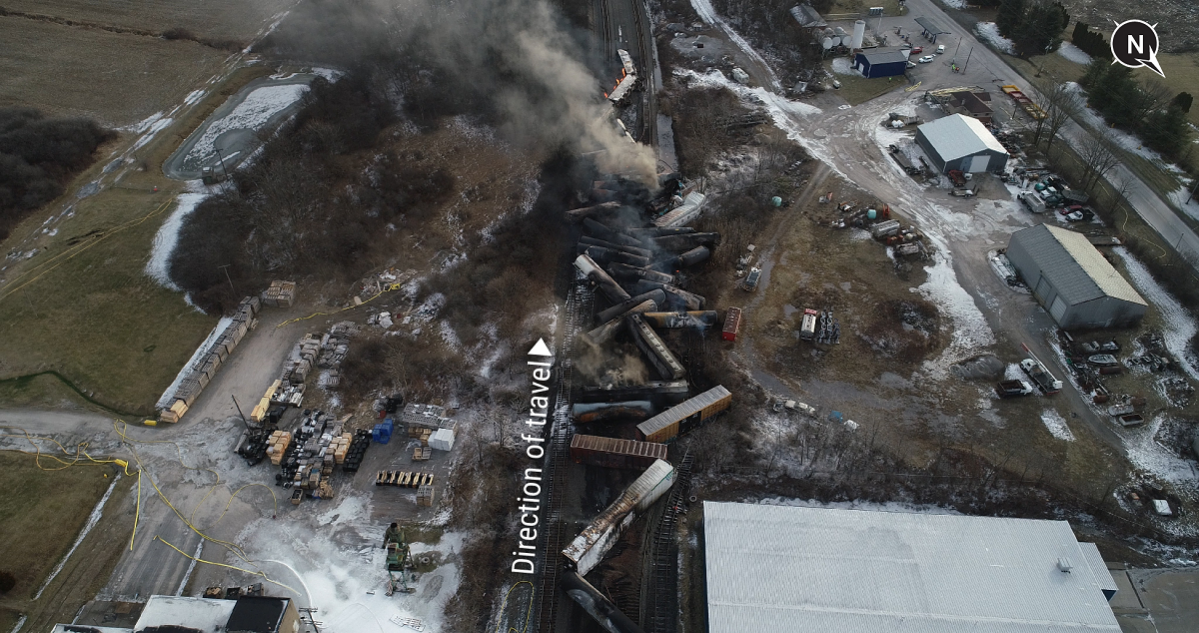
Aerial photograph of the train derailment site. Photo credit: National Transportation Safety Board [1].

In a now well-publicized event, a Norfolk Southern Railway train hauling 149 cars, 20 of which were tanker cars containing hazardous industrial chemicals, derailed in East Palestine, Ohio, in the evening hours of February 3, 2023 [1] (Figure 1). As highly toxic vinyl chloride (VC) was released into the air, soil, and water surrounding the crash site, evacuation orders, region-wide contamination fears, and a federal investigation ensued. The National Transportation Safety Board’s (NTSB) preliminary report identified an overheated wheel bearing as responsible for the derailment, which was deemed entirely preventable [1]. Norfolk Southern set a critical threshold for its hot bearing detectors at 93.33 °C above ambient temperature, so despite escalating readings, a critical warning was not transmitted until the faulty bearing’s temperature was 122.78 °C above ambient temperature [1]. Fires resulting from the accident burned for 2 days before containment. However, because of the continued temperature rise within VC-containing tankers, the concern for an ongoing VC polymerization reaction with an impending explosion was heightened. Consequently, a controlled venting and burning of VC from 5 tankers was undertaken on February 6, requiring the expansion of the evacuation zone to 2 miles [1]. Although the exact amount of VC spilled remains under investigation, the NTSB estimates 115,580 gallons were released [1].

VC is a manmade organochloride used in the production of plastics composed of polyvinyl chloride (PVC). Occupational, environmental, and accidental exposure to VC is associated with a range of health effects spanning multiple organ systems [2]. Although the emphasis of this review is on the dermatologic manifestations of VC exposure, the interconnectedness of these effects with toxicity to other organ systems must be acknowledged. The liver is identified as a major target organ; however, acute exposure–related illness along with chronic carcinogenic, immunologic, and neurologic effects are also widely characterized [2]. Acute exposure via inhalation causes respiratory irritation, headache, nausea, vomiting, dizziness, fatigue, weakness, and confusion. In high concentrations, exposure leads to nervous system depression, arrhythmias, coma, and death [2]. Importantly, prolonged exposure is linked to numerous cancers, namely hepatic angiosarcoma, hepatocellular carcinoma, and malignancies of the brain, lungs, skin, and hematopoietic system. Other chronic health effects include peripheral neuropathy, nephrotoxicity, immune disorders, steatohepatitis and cirrhosis, and acro-osteolysis (AOL) [2].

VC is transported as a liquefied gas under vapor pressure. Given its flammability, exposure to intense heat may result in container explosion, greatly magnifying dispersal during transportation and industrial accidents. Historically, VC exposure occurred among factory workers in direct contact with the chemical during PVC manufacturing. However, community exposures secondary to intentional industrial releases and accidental spills have occurred for decades and are well-reported [3] (Table 1). Despite these reports, VC exposure remains an underrecognized public health threat. Herein, this review emphasizes valuable insights into the serious health risks of VC exposure, with particular attention to cutaneous manifestations, and the broader impact of environmental toxin releases, particularly for vulnerable populations.

**Table 1:** VC^a^ chemical spills, industrial accidents, and environmental contamination in the United States.

Year	Location	Source	Type of release	Amount or scope of VC released
1964	Hebronville, Massachusetts	Thompson Chemical Company	Industrial facility fire and explosion	70 tons released [4].
1950s-1985	US Marine Corps Base Camp Lejeune, North Carolina	Multiple sources	Leaking underground storage tanks, industrial site spills, and contamination from waste disposal sites	Unknown amount released. One million military and civilian staff and their families were potentially exposed [5].
1983	Delaware City, Delaware	Stauffer Chemical Company and Formosa Chemical Corp	The plant was deemed a Superfund site due to contamination from earthen lagoons and pits used for the disposal of PVC^b^ waste and sludge	EPA^c^ testing demonstrated VC contamination of groundwater at 220 μg/L (limit 5 μg/L), which is used locally for drinking water and agricultural purposes. Soil contamination was also identified [6,7].
2004	Illiopolis, Illinois	Formosa Chemical Corp	Industrial plastics manufacturing facility fire and explosion	150,000 gallons were present on site; an estimated 6000 gallons were released [8].
2005	Point Comfort, Texas	Formosa Chemical Corp	Industrial plastics manufacturing facility fire and explosion	Unknown amount released [6].
2005	Delaware City, Delaware	Formosa Chemical Corp	Industrial plastics manufacturing facility release due to equipment design flaw	2500 pounds released [6].
2012	Paulsboro, New Jersey	Conrail Company	Train derailment–related spill	24,000 gallons released [3,9].
2013	Westlake, Louisiana	Axiall Chemical	Industrial facility fire	Unknown amount released [10].
2017	Houston, Texas	Multiple chemical plants	Flooding and damage to industrial facilities during Category 4 Hurricane Harvey	Unknown amount released [11].
2020	Westlake, Louisiana	Multiple manufacturing plants and industrial facilities including Westlake Chemical	Damage to industrial facilities during Category 4 Hurricane Laura	Unknown amount released. Possible VC release reported to US Coast Guard’s National Response Center. Westlake Chemical incurs facility damage and power outages and declares force majeure [12,13].
2022	Flint, Michigan	Lockhart Chemical	Manufacturing facility spill	Unknown amount released [14].
2023	East Palestine, Ohio	Norfolk Southern Railway	Train derailment–related spill	Unknown amount was released but estimated to be as much as 1.1 million pounds [15].

^a^VC: vinyl chloride.

^b^PVC: polyvinyl chloride.

^c^EPA: Environmental Protection Agency.

## Methods

To characterize the cutaneous effects associated with VC exposure, especially those resulting from industrial releases and accidental spills, a narrative review was conducted using a comprehensive approach to identify relevant evidence-based, peer-reviewed scientific sources, gray literature, and media reports. Searches were performed in reputable databases such as PubMed and Google, using predetermined keyword search terms related to VC, spills and releases, train derailment, cutaneous disease, public health, and vulnerable and marginalized populations. We recognize this nonsystematic approach has inherent limitations of content selection, such as the potential for subjectivity and bias [16], but given the paucity of recent scientific studies in the medical literature on this topic and the critical importance of considering investigations by and proceedings of federal agencies, commissioned governmental assessments, litigation documents, investigative journalistic reporting, and media accounts to the exploration of this issue, a narrative approach was pursued in order to more broadly capture the public health and equity implications of VC-related exposures.

## Results

### Production and Environmental Contamination in the United States

Production of VC began in the 1930s. Currently, 99% of VC is used to manufacture PVC, of which 7.2 million metric tons were produced in 2019 in the United States [2,17]. The largest concentration of facilities that currently produce, process, or use VC are located in Texas (12 sites), Louisiana (8 sites), and Kentucky and Ohio (3 sites each) [2]. These facilities house massive amounts of VC, with some authorized to store 500 million pounds [2]. Among US states, Louisiana ranks as a top producer of toxic wastes per capita, a fact which community activists have long decried as the source of disproportionate health impacts observed in residents living near the state’s many chemical plants [6]. Not surprisingly, the heavily industrialized corridor between New Orleans and Baton Rouge is known as “cancer alley” because of the region’s markedly higher rates of malignancy [18].

Under the Toxics Release Inventory (TRI) Program, the Environmental Protection Agency (EPA) tracks toxic chemicals that pose a threat to human health and the environment, including VC [2,19]. Manufacturing facilities are encouraged to first pursue source reduction of toxic waste; however, for generated waste, preferred management methods include recycling, treatment, and neutralization, and as a last resort, disposal or release into the environment. In 2021, approximately 428,500 pounds of VC were released into the environment based on mandatory reporting to the EPA under the provision of the TRI program [19].

While VC should volatilize to the atmosphere, its water solubility may allow leaching through soil and into groundwater prior to evaporation [2]. As a result, chemical releases and leaks can lead to extensive soil and groundwater contamination and are known to persist for years, as evidenced by enduring levels exceeding regulatory limits in groundwater wells. VC may also enter household air via volatilization from contaminated water that is heated and used for bathing, cooking, or laundry [20]. Consequently, populations residing in heavily industrialized areas surrounding plastic manufacturing plants may experience ongoing exposure to this carcinogen.

A particularly noteworthy example is the Axiall Chemical Plant in Westlake, Louisiana, which reportedly stores 100 million pounds of VC and has a long history of industrial accidents, fires, and major environmental violations [21]. EPA surveys identified contamination by hazardous substances, including VC, in sediments, surface waters, and the biota of the nearby Calcasieu Estuary. This prompted a federal lawsuit in 2021 against Axiall and 8 other petrochemical manufacturers under the Comprehensive Environmental Response, Compensation, and Liability Act “seeking reimbursement of response costs incurred or to be incurred for response actions taken at or in connection with the release or threatened release of hazardous substances at the Calcasieu Estuary” [22].

Beyond EPA-tracked and regulated toxic releases, uncontrolled or illegally released hazardous substances as a result of acute chemical incidents also pose a significant public health threat. According to the last report by the National Toxic Substance Incidents Program, 14,175 acute chemical incidents, including VC releases, were documented in the United States in 2013 with 64% associated with fixed facility releases and 36% attributed to transportation-related releases [23]. Volatilization and chemical spills account for the 2 most common release types, with volatilization contributing to 54.4% of injuries and 27% of fatalities. Chemicals that readily volatilize, such as VC, have the potential to quickly expose large numbers of people before evacuation or shelter-in-place orders are issued, explaining why a VC release from a train derailment caused a mass injury event that year [23]. Alarmingly, 1044 train derailments occurred in the United States in 2022 [24]. The sheer number of acute chemical incidents reported annually and the magnitude of the recent VC release in Ohio clearly highlight that future response plans must reduce public exposure following chemical incidents.

### The Dermatological Effects of VC Exposure

The full magnitude of health impacts resulting from the recent VC spill in Ohio may not be realized for years to come. However, acute health concerns have already emerged. For example, many residents have anecdotally noted skin “rashes” in the aftermath of the spill [25]. Acute dermatological manifestations were similarly reported in 5.1% of patients presenting to emergency rooms in 2012 following VC release from a New Jersey train derailment [26]. Cutaneous toxicities resulting from chemical spills may be significant because, relative to other organs, the skin has the greatest surface area in contact with a given environmental exposure, underscoring the vital need for dermatologists and other health professionals to recognize the potential acute and delayed cutaneous manifestations of VC exposure following industrial accidents and chemical spills.

Most existing reports focus on chronic occupational exposure and predate 1995. Accordingly, the medical literature appears to have an incomplete understanding of the impacts of acute and persistent environmental VC exposure following toxic environmental release events. As such, health care providers may struggle to identify and treat the immediate and long-term effects of VC exposures, suggesting that further studies are needed to fully elucidate the health risks posed by VC in these situations. Despite existing limitations, evidence suggests that VC exposure causes short- and long-term skin-related manifestations, which we describe in detail herein [27,28].

Cutaneous exposure to VC may cause contact dermatitis, while rapid evaporative cooling of liquid VC on the skin may result in frostbite with erythema, blistering, and desquamation [2]. Individuals whose clothing or skin is contaminated with pressurized liquid VC can secondarily contaminate others via direct contact or through airborne release of the chemical [29].

VC exposure may lead to the development of vinyl chloride disease, which is characterized by the triad of Raynaud symptoms, sclerodermatous skin changes, and lytic bone lesions known as AOL [27]. Variable phenotypic presentations are observed after VC exposure with 10% displaying cutaneous sclerodermoid changes, 6% demonstrating Raynaud phenomenon, and 3% developing AOL [30].

The scleroderma-like changes observed in VC exposure are characterized by unique clinical features that help in distinguishing this entity from primary systemic sclerosis (SS). Namely, the cutaneous lesions in VC-associated disease primarily affect the dorsal hands and forearms and present as papules, nodules, and plaques with associated pruritus and hyperhidrosis. Additionally, coarsening of the skin on the forehead and cheeks may occur, whereas microstomia, matted telangiectasias, and calcinosis—classic features of limited cutaneous SS—are generally absent. Clubbing and fingertip shortening accompanied by lytic lesions in the distal phalanges, the key features of AOL, typify VC-associated scleroderma rather than the more common distal tufting observed in SS [3,30]. VC-induced scleroderma is typically seronegative for antinuclear, anticentromere, and anti-Scl-70 antibodies [2,30]. Histology of VC-associated sclerodermatous lesions has both shared and distinct features when compared to SS. In both conditions, hyperkeratosis, epidermal atrophy, and thickening of dermal collagen bundles with disorganization and fragmentation of elastic fiber are observed. Interestingly, these histological changes are also seen in clinically uninvolved skin in one-quarter of individuals exposed to VC, suggesting genetic factors likely play a role in the phenotypic presentation of VC disease among those exposed [30]. Notably, adnexal structures, specifically eccrine glands, are often atrophic in classic SS but preserved in VC disease [30].

Raynaud syndrome, a dominant sign resulting from VC exposure, may be severe and accompanied by trophic changes and digital ulceration. Progressive bone resorption and deformity of the distal phalanges in AOL may damage underlying vasculature, causing secondary skin and nail changes, including clubbing and nail dystrophy [2]. In one report, one-third of workers in contact with VC developed Raynaud syndrome, with symptoms persisting for ≥15 years after exposure [31]. In classic Raynaud, chronic abnormal vasospastic responses produce microangiopathic changes over time; however, the pathomechanisms underlying VC-induced Raynaud disease remain incompletely understood. Hand angiographic findings in patients with VC-associated Raynaud disease universally identified arterial abnormalities including vascular occlusion, stenosis, thread-like narrowing, and development of collateral circulation [32]. Although microvascular alteration is a contributing factor, it does not explain the entire picture. For example, relative to controls in one study, the VC-exposed cohort exhibited persistent nail fold capillary changes and carried a significantly higher prevalence of Raynaud symptoms, yet those symptoms were not statistically related to the degree of capillaroscopic modification [31]. Functional proof tests provide further insight into potential mechanisms driving chronic cutaneous symptoms of VC exposure. Abnormal plethysmography, vibration perception, and cooling tests in exposed individuals suggest that VC-induced cutaneous vascular and peripheral neuropathic damage play a role [28].

Additionally, evidence suggests that VC exposures alter immunologic function and may lead to the observed autoimmune presentations observed clinically. Prior studies in VC-exposed workers and animal models dating back decades demonstrate a range of responses, including B-cell proliferation, hypergammaglobulinemia, oxidative stress, elevated complement levels, and production of proinflammatory cytokines (tumor necrosis factor-α and interleukin-1β, -6, and -8) [2]. Interestingly, similar cytokine profiles are observed after activation of cutaneous xenobiotic receptors and transcription factors, such as the aryl hydrocarbon and pregnane-X receptors, in response to binding by environmental pollutants including particulate matter, polycyclic aromatic hydrocarbons, and benzene [33]. Unfortunately, little research has focused on elucidating the molecular pathways responsible for cutaneous toxicities to VC, highlighting the large gaps in our understanding of the pathogenesis.

However, studies suggest that VC-induced Raynaud disease may, in part, be triggered by immune complex deposition. A study examining 22 patients with a history of VC exposure, who subsequently developed Raynaud disease, detected cryoglobulins in 81% of sera samples with cryoglobulinemia occurring, in part, due to hyperactivation of humoral responses following the exposure [34]. Disturbances in humoral immunity are likely to also play a role in the systemic effects of VC, including hepatotoxicity and neurologic disease [2]. Genetic factors, such as human leukocyte antigen (HLA)-B8 and HLA-DR3, correlated with severe scleroderma-like disease following VC exposure, whereas the HLA-DR5 haplotype may also increase the susceptibility risk of Raynaud disease [2]. Moreover, polymorphisms in the M-1 and T-1 genes encoding glutathione-S-transferases, which are involved in VC-metabolism, are linked to the development of Raynaud [2].

Tissue fibrosis within the liver, skin, lungs, and kidneys is a well-reported consequence of VC toxicity, but the pathomechanisms responsible for inducing cutaneous fibrosis remain poorly understood. Studies of VC-associated hepatic, renal, and pulmonary fibrosis demonstrate upregulation of plasminogen activator inhibitor-1, transforming growth factor-β, platelet-derived growth factor, vascular endothelial growth factor, and connective tissue growth factor [35-37]. Since these growth factors are implicated in the pathogenesis of SS, we purport this serves as a likely corollary for cutaneous fibrosis observed after VC exposure [38]. However, further research is needed to fully elucidate the immunological, vascular, and neuropathic factors in VC-mediated dermatological disease.

The carcinogenicity of VC was established in the 1960s [39]. Evidence suggests a possible association of VC with cutaneous malignant melanoma and squamous cell carcinoma. Immune dysregulation and genetic-environmental interactions mediated by CYP2E1 metabolism lead to reactive intermediates that induce tumor suppressor gene mutations in *ras* and *p53*, which are implicated in cutaneous carcinogenesis [40-42]. Genetic polymorphisms in CYP2E1 and glutathione-S-transferases increase VC-induced mutations, allowing for the potential identification of high-risk individuals [42]. Moreover, geographic mapping of industrial releases and accidents may help to identify high-risk populations who should receive serial monitoring for cutaneous malignancy [18].

Additionally, cutaneous stigmata of chronic hepatic injury, including spider angiomas, palmar erythema, xanthelasma, jaundice, and pigmentary changes may occur, as well as thrombocytopenic purpura as a consequence of portal hypertension-induced splenomegaly and platelet sequestration [28]. One report described VC-associated purpura in the setting of lymphohistiocytic infiltrate within dermal arterioles on histopathology, circulating immune complexes, and anti–smooth muscle autoantibodies on laboratory work-up, suggesting immunological dysfunction was playing an etiological role [43].

When assessing dermatological effects, the volume of VC released into the environment along with the duration, intensity, and route of exposure (eg, direct skin contact, inhalation, and ingestion) must be considered. Variability in individual susceptibility to VC toxicity based on overall medical status and underlying genetic predisposition should dually be considered. For example, those with preexisting atopy, autoimmunity, vascular injury, or polymorphisms that increase VC-induced carcinogenic mutations may be at increased risk of cutaneous disease [42]. Finally, it is important to account for the possibility that VC may synergistically interact with other toxins released during spills and industrial accidents as this could lead to novel health effects.

### Treatment and Prevention of VC-Induced Disease

To minimize the risk of long-term health issues, prompt medical evaluation is crucial in cases of VC exposure. Dermatologists, public health experts, and frontline health workers play a critical role in identifying and treating exposure-related skin conditions as well as detecting individuals at risk of internal sequelae. Following acute exposure, the priority is decontamination by removing saturated clothing and cleansing exposed skin and hair with soap and water to halt further exposure. Notably, children are more susceptible to skin absorption than adults due to a higher surface area to body weight ratio [29]. Frostbite injury is treated by rewarming affected areas in a hot-water bath (38.89-42.22 °C), and chemical burns are treated as thermal burns [29]. With no established treatments for VC-associated cutaneous manifestations, the efficacy of standard therapeutic agents—such as calcium channel blockers, α-1-adrenergic receptor antagonists, angiotensin receptor blockers, topical nitrates, fluoxetine, phosphodiesterase inhibitors, endothelin-1 receptor antagonists, or antiplatelet agents for Raynaud symptoms and methotrexate, mycophenolate mofetil, cyclophosphamide, D-penicillamine, intravenous immunoglobulin, hematopoietic stem cell transplantation, tocilizumab and rituximab for VC-induced SS—remains uncertain [44,45]. Thus, further investigation into effective and safe therapeutic approaches in VC-associated autoimmune diseases is warranted.

Because VC persists in environmental reservoirs for years, clinicians should remain alert to long-term manifestations arising from chronic exposure. The Occupational Health and Safety Administration [46] recommends annual medical surveillance for any exposure over the minimal risk level of 0.5 ppm. However, no standardized guidelines currently exist for surveillance and long-term management of chronic dermatological conditions. Moreover, VC exposure may affect other organ systems with dermatological conditions often arising as a cutaneous manifestation of systemic disease. Therefore, we also advocate for longitudinal screening for dermatological manifestations as a component of required medical surveillance and as a compensated benefit to victims of industrial accidents or chemical spills. However, we recognize that currently available data is insufficient to mandate longitudinal dermatology screening, underscoring a key evidence gap. Consequently, emergency room physicians, frontline and public health professionals, and primary care physicians play a crucial role in screening individuals with VC exposure for health impacts both acutely and longitudinally, underscoring the need to recognize the associated cutaneous manifestations of VC exposure and generate referrals to dermatologists for appropriate evaluation and treatment.

With limited treatment options for VC-related exposure, prevention is paramount. Public health officials play an integral role in preventing and mitigating chemical exposure–related health conditions through regulatory oversight and health policy implementation. While the precise cause of the Ohio derailment remains under investigation, decades of divestment in government spending for maintenance of infrastructure, including transportation networks, coupled with industry participation in the EPA’s peer review process for scientific assessments in which disease thresholds and exposure limits to toxic substances are established, widespread environmental deregulation, and successful lobbying against broad definitions for high-hazard flammable trains and implementation of electronically controlled pneumatic brakes on trains transporting hazardous materials contribute collectively to the likelihood of future disasters [39,47,48]. Moreover, the increasing frequency and intensity of extreme weather events due to climate change amplifies the risk of secondary disasters due to toxic environmental releases resulting from damage to industrial facilities, such as those documented following Hurricanes Katrina, Harvey, and Laura [49,50]. This further emphasizes the need to ensure the integrity of industrial infrastructure, climate resiliency, disaster management and response, improved railroad safety, safety inspections for transportation of hazardous materials, enforcment of safety regulations, mandatory release notifications, ongoing monitoring of chemical spills and releases, and development of public health campaigns to promote education on chemical exposures.

### Environmental Justice and Public Health Implications

With respect to health equity, dermatologists, public health professionals, and primary care providers should recognize that hazardous chemical exposures exacerbate existing health disparities among marginalized populations. In the United States, most railroads traverse neighborhoods of lower socioeconomic status and racially divide cities, while fenceline communities in industrial zones are predominantly Black [6,51]. In East Palestine, Ohio, the median household income was US $49,407 in 2020, versus US $64,994 nationally [52]. Beyond overwhelming concerns for health impacts, the average cost to evacuate after a disaster is US $5000 per family [53]. Consequently, the financial ramifications on this community are significant as the derailment forced residents from their homes, shuttered businesses, contributed to lost wages, sickened livestock, and portends looming medical bills [54]. Disproportionate exposures to toxic pollution are well-documented. Nationally, hazardous waste sites and industrial facilities are more often sited in proximity to Black communities and those of lower socioeconomic status, a historic legacy of discriminatory practices such as redlining [6,55]. However, such practices persist. Due to existing disparities in the granting of permits by state regulators in Louisiana, where a large number of PVC plants operate, industrial emissions are 7- to 21-fold higher in Communities of Color. Not surprisingly, these same communities have higher rates of cancer and respiratory disease and overall greater health impacts from pollution [6]. The dermatologic manifestations of environmental pollution are well described in the medical literature. Therefore, it is imperative to recognize that existing disparities in skin health, including higher incidence of cutaneous disease and limited access to health care, among lower-income and minority populations are further amplified by the unequal impact of hazardous exposures in these groups [56].

## Discussion

The 2023 Ohio train derailment has renewed the spotlight on the health consequences arising from chemical exposures following environmental releases. Because most existing literature on VC is decades old and focuses primarily on occupational exposure, accurately assessing potential risks to public health associated with chemical spills, including identification and treatment of exposure-related dermatological conditions, is challenging for health professionals. Despite these limitations, evidence suggests acute dermatological manifestations may include contact dermatitis and frostbite. Notable subacute-to-chronic manifestations include Raynaud disease, acral sclerodermatous changes, AOL, skin cancer, and cutaneous stigmata of liver disease. In addition to individual susceptibility, factors that may influence severity include duration, intensity, and route of exposure.

We advocate for the consideration of viable alternatives to plastic products requiring the use of VC in their production. Examples of such alternatives include glass, ceramics, and linoleum. European nations have implemented restrictions and outright bans on VC use, signaling growing recognition of the importance of risk reduction associated with this hazardous chemical [57]. Furthermore, greater awareness of the dermatological effects of environmental chemical exposures is needed, with special consideration for marginalized and vulnerable populations, underscoring the urgency for improved public health measures, legal and regulatory frameworks, and policies to ensure timely and effective responses to and prevention of chemical spills and other environmental disasters.

## Abbreviations

AOL: acro-osteolysis
EPA: Environmental Protection Agency
HLA: human leukocyte antigen
NTSB: National Transportation Safety Board
PVC: polyvinyl chloride
SS: systemic sclerosis
TRI: Toxics Release Inventory
VC: vinyl chloride
